# Effectiveness of the MULTIPAP Plus intervention in youngest-old patients with multimorbidity and polypharmacy aimed at improving prescribing practices in primary care: study protocol of a cluster randomized trial

**DOI:** 10.1186/s13063-022-06293-x

**Published:** 2022-06-09

**Authors:** Isabel del Cura-González, Juan A. López-Rodríguez, Francisca Leiva-Fernández, Luis A. Gimeno-Feliu, Victoria Pico-Soler, Mª. Josefa Bujalance-Zafra, Miguel Domínguez-Santaella, Elena Polentinos-Castro, Beatriz Poblador-Plou, Paula Ara-Bardají, Mercedes Aza-Pascual-Salcedo, Marisa Rogero-Blanco, Marcos Castillo-Jiménez, Cristina Lozano-Hernández, Antonio Gimeno-Miguel, Francisca González-Rubio, Rodrigo Medina-García, Alba González-Hevilla, Mario Gil-Conesa, Jesús Martín-Fernández, José M. Valderas, Alessandra Marengoni, Christiane Muth, J. Daniel Prados-Torres, Alexandra Prados-Torres, MULTIPAP PLUS Group, Francisco Javier Orellana-Lozano, Jesús Sepúlveda-Muñoz, Rafael Sánchez-Jordán, Amparo Escobar-Pérez, Concepción Rodríguez-García, Trinidad Peñuela-Ruiz, José Antonio Navarro-Martín, María Rosario Rodríguez-Rivera, Yolanda Aguilar-Heredia, Antonio Ignacio Martínez-Sarmiento, Beatriz Pascual-de-la-Pisa, María José García-Lozano, Alejandro García-Carrera, Noelia Juan-Tordesillas, María Isabel Márquez-Chamizo, José Manuel Navarro-Jiménez, María Carmen Ruiz-Ciudad, Rubén Luciano Vázquez-Alarcón, María Isabel Navarro-Gallego, Leovigildo Ginel-Mendoza, José María Ruiz-San-Basilio, Elena Barceló-Garach, Elisa María Alcantarilla-Reyes, Marta Álvarez de Cienfuegos Hernández, Irene Martínez-Ríos, Laura Orellana-Martín, María Dolores Merino-Moyano, Nuria Segura-Domínguez, María Cristina Moral-Merchán, Esther Martín-Aurioles, María Inmaculada Rodríguez-González, Sylvia Hazañas-Ruiz, Eva Noelia Gallego-Castillo, Esperanza Mora-García, Estefanía Cámara-Sola, Sergio Fons-Cañizares, María Paz Ortigosa-Arrabal, Teresa Quesada-Fernández, Silvia Rodríguez-Moreno, Ana Sánchez-Silvestre, María Jesús Torrubia-Fernández, María José González-Vega, María Victoria Almagro-Martín-Lomeña, Caridad Serrano-González, José Leiva-Fernández, Virginia Castillo-Romero, Ana María Fernández-Vargas, Francisco José Serrano-Guerra, Gabriel Francisco Narbona-Carrión, Hervé Michel-Bertevas, Rafael Ángel Maqueda, Miguel Domínguez-Santaella, Nuria García-Agua-Soler, Maria del Pilar Barnestein-Fonseca, María Begoña Abadía-Taira, Carmen Sánchez-Celaya-del-Pozo, Ana Carmen Giménez-Baratech, Lara Sanz-Burgos, Mercedes Abad-Royo, Carmen Camats-Franco, José Manuel Cortés-Pellicer, Paula Herrero-Solsona, Aida Moreno-Juste, Miguel Guiu-Campos, Nima Peyman-Fard-Shafi-Tabatabaei, Ma Teresa Delgado-Marroquín, Mercedes López-Echevarría, Jonás Carmona-Pírez, Fernando Barrera-Linares, Sandro Daniel Carrillo-Soria, Ana Belén Esteban-Gimeno, Beatriz López-Alonso, Anabel Hernández-Bono, Enrique Martínez-Ayala, Adriana Martínez-Manero, Raquel Martínez-Sánchez, Yolanda Naya-Mateu, María Lourdes Clemente-Jiménez, María Paz Leon-Martínez, Liliana Mahuela, Ma Rosario Sanjuan-Cortés, Elisa Pilar Salazar-González, Ma Elena Charte-Alegre, Ma Jesús Mur-Lazuela, Mónica Pascual-Franco, Pilar Arizon-Deza, Carmen García-Gutiérrez-Muñoz, Teresa García-Ruiz, Gloria Navarro-Aznárez, Carlos Alcober-Pérez, María Paz Navarro-Tausiet, Ma Elena Lacasa-Serrano, Ana Cristina Maza-Invernón, Jaime Peleato-Sánchez, José Miguel Buñuel-Granados, Ainara Alonso-Valbuena, Mónica Lasheras-Barrio, Isabel Ibarrondo-Fernández-Ladreda, Rosa Ma López-Aylon, María José Rodríguez-Fabre, Isabel Rubio-Gutiérrez, Selma Valverde-Aranda, Ana Cristina Bandrés-Liso, Antonio Poncel-Falcó, Kevin Bliek-Bueno, Mabel Cano-del-Pozo, Mercedes Clerencia-Sierra, Jesús Díez-Manglano, Inmaculada Guerrero-Fernández-de-Alba, Ignatios Ioakeim-Skoufa, Javier Marta-Moreno, David Santos-Muñoz, María Elisa Viñuela-Benéitez, María De Los Angeles Miguel-Abanto, Francisca García-De-Blas, Juan Carlos García-Álvarez, Sonia Redondo-de-Pedro, Carlos Fernando González-García, Carolina Peláez-Laguno, Esther Gomez-Suarez, Fernanda Morales-Ortiz, Isabel Ferrer-Zapata, Yolanda Beatriz Sánchez-Fernández, Yolanda Fernández-Fernández, Esther Barrio-Higelmo, Eva María Rioja-Delgado, Irina Lopez-Larrayoz, María Luz Seara-Lozano, Julio Cesar Fernández-Sánchez, María Teresa San-Miguel-Marinero, María Jesus Fidalgo-Baz, Sara Ares-Blanco, Jorge Ignacio Gómez-Ciriano, José Damián Garcés-Ranz, Laura Santos-Franco, María Celeste García-Galeano, Raquel Mateo-Fernández, Sara Morcillo-Cebolla, Tomás Rossignoli-Fernández, Jorge Olmedo-Galindo, Marta Pinel-González, Rosa María Redondo-Romero, Adnaloy Helena Estrada-Leon, Belén Muñoz-Gómez, Blanca Sanz-Pozo, Claudia López-Marcos, Enrique Rodríguez-De-Mingo, Juan Carlos Moreno-Fernández, Luis Enrique Morales-Cobos, María Del Prado Garcia-Garcia-Alcañiz, Marisol Lorenzo-Borda, Vera González-García, María Del Pilar Muñoz-Molina, Yasmin Drak-Hernández, Alejandro Rabanal-Basalo, Ana María Abad-Esteban, María De Los Ángeles Rollan-Hernández, Mónica Fuster-Tozer, Raquel Carretero-Ramos, Rebeca Mielgo-Salvador, Ana Sosa-Alonso, Carmen María Muros-Muñoz, Jeannet Dolores Sánchez-Yépez, María Cristina Cáceres-Cortés, María Paloma Morso-Peláez, María Pastor-Estebanez, Mercedes Fernández-Girón, Antonia Pérez-De-Colosia-Zuil, Esteban Pérez-Gutiérrez, Isabel Tejero-García, Jaime Innerarity-Martínez, Mar Álvarez-Villalba, Margarita Gómez-Barroso, María Del Mar Escobar-Gallegos, María Jesus Bedoya-Frutos, Marta Inmaculada Del-Olmo-Ribagorda, Petra María Cortés-Durán, Pilar Tardáguila-Lobato, Raquel Yolanda Terrón-Barbosa, Antonio Ramos-Blanco, Aránzazu López-Villalvilla, Beatriz Cinta-Bella, Cristian Varela-Varela, Francisca Garcia-Rodriguez, Gema María Saiz-Ladera, Guillermina López-Fernández, Lourdes Orozco-Barrenechea, María Begoña Zafra-De-Gea, Nuria García-Arpa, Tamara Ewa-Barnas, Ana Isabel Carbonero-Martín, María José Rojas-Giraldo, Alberto Cotillas-Rodero, Beatriz López-Serrano, María Del Carmen Rodriguez-Fernández, Carmelina Sanz-Velasco, Jose Ignacio Aza-Pascual-Salcedo, Carolina Lopez-Olmeda, Estrella Gutiérrez-Ocana, Raquel García-Ocaña, Teresa Sanz-Cuesta, Ricardo Rodríguez-Barrientos, Milagros Rico-Blázquez, Ma Gloria Ariza-Cardiel, Angel Mataix-San-Juan, Marta Alcaraz-Borrajo, Mercedes Rumayor-Zarzuelo, Luis Sánchez-Perruca, Amaya Azcoaga-Lorenzo, Virginia Hernández-Santiago, Rafael Rotaeche-del-Campo

**Affiliations:** 1 Research Unit, Primary Care Assistance Management, Madrid Health Service, Madrid, Spain; 2 Department of Medical Specialties and Public Health, School of Health Sciences, Rey Juan Carlos University, Madrid, Spain; 3 Research Network on Health Services in Chronic Diseases REDISSEC-ISCIII, Madrid, Spain; 4 Instituto de Investigación Sanitaria Gregorio Marañon (IiSGM), Madrid, Spain; 5 Research Networks Health Outcomes-Oriented Cooperative on Chronicity, Primary Care and Health Promotion RICORS RICAPPS, ISCIII, Madrid, Spain; 6 Ricardos General Health Center, Madrid Health Service, Madrid, Spain; 7 Multiprofessional Family and Community Care Teaching Unit of the Málaga-Guadalhorce Health District, Málaga, Spain; 8 Biomedical Research Institute of Malaga (IBIMA), Málaga, Spain; 9 University of Malaga, Malaga, Spain; 10 San Pablo Primary Care Health Centre, Aragon Health Service, Zaragoza, Spain; 11 University of Zaragoza, Zaragoza, Spain; 12 EpiChron Research Group, Aragon Health Sciences Institute (IACS), IIS Aragón, Miguel Servet University Hospital, Zaragoza, Spain; 13 Torrero-La Paz Primary Care Health Centre, Aragon Health Service (SALUD), Zaragoza, Spain; 14 La Victoria Health Center, Málaga-Guadalhorce Health District, Málaga, Spain; 15 Primary Care Pharmacy Service Zaragoza III, Aragon Health Service, Zaragoza, Spain; 16 Health Center Campillos, Malaga North District (Antequera), Málaga, Spain; 17 Primary Health Care Research and Innovation Foundation FIIBAP, Madrid, Spain; 18 Delicias Sur Primary Care Health Centre, Aragon Health Service (SALUD, Zaragoza, Spain; 19 Preventive Medicine Service, University Hospital Alcorcon Foundation, Madrid, Spain; 20 Multiprofessional Family and Community Care Teaching Unit West, Madrid, Spain; 21 Department of Family Medicine, National University Health System, Singapore, Singapore; 22 Department of Clinical and Experimental Sciences, University of Brescia, Brescia, Italy; 23 Department of General Practice and Family Medicine, Medical Faculty OWL, University of Bielefeld, Bielefeld, Germany

**Keywords:** Multimorbidity, Patient-centred care, Polypharmacy, Medication reconciliation, Decision-making, Computer-assisted, Primary health care, Cluster randomized controlled trial

## Abstract

**Background:**

The progressive ageing of the population is leading to an increase in multimorbidity and polypharmacy, which in turn may increase the risk of hospitalization and mortality. The enhancement of care with information and communications technology (ICT) can facilitate the use of prescription evaluation tools and support system for decision-making (DSS) with the potential of optimizing the healthcare delivery process.

**Objective:**

To assess the effectiveness and cost-effectiveness of the complex intervention MULTIPAP Plus, compared to usual care, in improving prescriptions for young-old patients (65-74 years old) with multimorbidity and polypharmacy in primary care.

**Methods/design:**

This is a pragmatic cluster-randomized clinical trial with a follow-up of 18 months in health centres of the Spanish National Health System. Unit of randomization: family physician. Unit of analysis: patient.

**Population:**

Patients aged 65–74 years with multimorbidity (≥ 3 chronic diseases) and polypharmacy (≥ 5 drugs) during the previous 3 months were included.

**Sample size:**

*n* = 1148 patients (574 per study arm).

**Intervention:**

Complex intervention based on the ARIADNE principles with three components: (1) family physician (FP) training, (2) FP-patient interview, and (3) decision-making support system.

**Outcomes:**

The primary outcome is a composite endpoint of hospital admission or death during the observation period measured as a binary outcome, and the secondary outcomes are number of hospital admission, all-cause mortality, use of health services, quality of life (EQ-5D-5L), functionality (WHODAS), falls, hip fractures, prescriptions and adherence to treatment. Clinical and sociodemographic factors will be explanatory variables.

**Statistical analysis:**

The main result is the difference in percentages in the final composite endpoint variable at 18 months, with its corresponding 95% CI. Adjustments by the main confounding and prognostic factors will be performed through a multilevel analysis. All analyses will be carried out in accordance to the intention-to-treat principle.

**Discussion:**

It is important to prevent the cascade of negative health and health care impacts attributable to the multimorbidity-polypharmacy binomial. ICT-enhanced routine clinical practice could improve the prescription process in patient care.

**Trial registration:**

ClinicalTrials.gov NCT04147130. Registered on 22 October 2019

**Supplementary Information:**

The online version contains supplementary material available at 10.1186/s13063-022-06293-x.

## Contributions to the literature

- There is lack of evidence about the effectiveness of interventions in primary care to prevent the cascade of negative health care impacts attributable to the multimorbidity-polypharmacy binomial.
- There are conflicting results on the effectiveness of decision support systems (DSS) to improve medication appropriateness. MULTIPAP-PLUS complex intervention combines a family physician training, a specific type of FP-patient interview, and a DSS to help family physicians.
- Medical record-implemented DSS could be a tool of great potential, and a co-designed DSS has been adapted to the conditions of clinical practice inside case report forms.

## Background

The progressive ageing of the population leads to an increase in multimorbidity, defined as ≥ 2 concurrent chronic medical conditions or ≥ 3 if a more specific threshold is considered to identify patients with complex health needs [1, 2]. In Spain, the average number of chronic problems in individuals > 75 years of age is 3.2; however, these patients constitute only part of the population with multimorbidity; among so-called older adults (65–74 years), it is 2.8 [3]. The importance of multimorbidity lies in its progressive increase with age [3] and its negative impact on health, reducing the quality of life and functional capacity of those who suffer from it. Likewise, it is associated with an increase in polypharmacy, hospitalizations, surgical complications, and mortality and, consequently, with a greater use of health services and therefore greater costs for health systems [4–7].

Within the framework of multimorbidity, polypharmacy acquires special importance. It is defined as the simultaneous consumption of ≥ 5 drugs and is considered the main determinant of potentially inappropriate prescription (PIP) in the elderly; this term encompasses excessive, incorrect, and insufficient prescriptions [8, 9]. Currently, it is estimated that 40% of the elderly population are polymedicated, having 36.5% of them PIP. This entails an increased risk of drug interactions and adverse drug reactions and therefore a low adherence and underuse of necessary treatments [10]. As people age, their risk of hospitalizations increases, as does their risk of fractures and therefore morbidity [11, 12] and mortality [7, 13]. It is estimated that 35% of elderly polymedicated patients in primary care (PC) develop some adverse effects, with 48.2% of adverse effects related to medication use and 59.1% of these being preventable [14, 15].

To quantify and reduce PIPs in complex patients, various measurement methods have been proposed: explicit, based on the properties of the drugs (BEERS and STOPP/START criteria), and implicit, based on the clinical judgement of the physician, who considers the overall situation of the patient and if the prescription responds to an indication or need, with the Medication Adequacy Index (MAI) being the most accepted method [16–18]. On the other hand, to improve polypharmacy, interventions at various scales have been proposed, for example (a) the professional level (educational programmes for prescribers or consumers); (b) the organizational level (use of electronic medical records, feedback to reduce pharmacological interactions, continuous review of medications, and decision support systems in the care process—DSS); (c) the patient level (education on the use of medications and treatment objectives); and (d) the financial level (prescribed incentives and regulatory interventions) [19].

In recent years, the management of patients with multimorbidity has been studied to provide evidence in the context of European health systems [20]. Spain has implemented the MULTIPAP intervention [21, 22], a complex intervention in the elderly population with multimorbidity and polypharmacy. The MULTIPAP intervention based on the Ariadne principles, which includes international guidelines for the treatment of patients with multimorbidity [21, 23], is effective in improving the appropriateness of medication at 12 months, although we should be cautious in the interpretation of the results given the paucity of evidence for the clinical benefit of the observed change in MAI. The MULTIPAP formative intervention provides clues for approaching this type of patient and identifies areas of improvement in training content. Along these lines, a Cochrane systematic review [24] that evaluates interventions for the management of patients with multimorbidity supports the need to generate more evidence to determine what specific training physicians need in the approach of these patients [25].

In this way, the incorporation of Information and Communication Technologies (ICT) in patient care is an opportunity to introduce medication evaluation tools such as DSS to optimize the care process. The DSS are considered a promising technology that improves medical care and the quality of prescription and reduces prescribing errors. However, more quality studies are needed to establish evidence of its effectiveness and cost-effectiveness in conditions of routine clinical practice [26, 27]. At the European level, some initiatives have been launched that attempt to overcome these limitations [28].

Among the tools available in Spain, CheckTheMeds® [29, 30] is an DSS designed as a health care tool that globally processes demographic, clinical and pharmacological data to detect issues related to medications (underdosing, overdosing, duplicates, therapeutic inertia, allergy alerts, drug-drug and drug-disease interactions, including inappropriate drug criteria, etc.). Its recommendations are based on the best available evidence. This tool has already been incorporated into some hospital pharmacy and PC services to generate treatment review reports that are subsequently sent to doctors. In our group within the MULTIPAP intervention, we tested the tool, offering proposals for improving and establishing a framework of collaboration with the platform that allows the use of its full potential for research studies in the field of PC [31, 32]. After the experience with the MULTIPAP project, the MULTIPAP Plus intervention was developed, introducing organizational measures that family physicians (FPs) have identified as essential. These include the inclusion of a DSS in the care process and its link with clinically relevant processes for patients, as well as the study of the economic costs involved in these clinical processes in the health system.

Although DSS may reduce PIPs, interventions in PC focused on patients with multimorbidity and polypharmacy with patient-relevant outcomes as hospital admission, mortality and quality of life between others are a priority, and several systematic reviews agree on the importance of implementing well-designed RCTs that incorporate outcome variables of clinical relevance for the patient, as well as the socioeconomic impact [24, 33].

## Aim

### Primary

The main objective is to evaluate the effectiveness of the complex intervention MULTIPAP Plus, in improving prescriptions to a young-old population (65–74 years) with multimorbidity and polypharmacy measured with a composite endpoint of hospital admission or death at 18 months, compared with usual care.

### Secondary

1. To evaluate the effectiveness of MULTIPAP Plus, measured as hospitalizations and/or all-cause mortality compared to those for routine practice at 12 months, as well as drug safety, use of services, quality of life of the patient measured with the EQ-5D-5L, disability, number of fractures, and adherence to treatment.
2. To study the cost-utility ratio for the intervention compared to usual care.
3. To describe the usability, adherence, and satisfaction with the DSS CheckTheMeds®.

## Methods

### Design

Pragmatic parallel two-arm, superiority, community-based cluster-randomized controlled clinical trial with a follow-up of 18 months. The unit of randomization is the FP, and the unit of analysis is the patient. The cost-utility study will be carried out from the perspective of the Spanish National Health System with a time horizon of 2 years.

CONSERVE-SPIRIT has been used for reporting trial protocol instead of SPIRIT since MULTIPAP Plus had been suspended because of COVID-19 from March 2020 to May 2021 and methodologic modifications were made (Additional file 1: CONSERVE-SPIRIT checklist; Additional file 2: SPIRIT Trial Modifications in Extenuating Circumstance). This situation has been notified and updated in the trial registry.

### Scope of study

The scope of study is primary care within the Spanish National Health System. The Spanish National Health System provides first contact, comprehensive, continuous, coordinated care (which is free at the point of care) to define a population served by primary care centres. Patients have named family physicians who are responsible for delivering and coordinating their care.

### Study population

The study population includes patients between 65 and 74 years with multimorbidity and polypharmacy attending in primary care health centres in three autonomous communities Aragón, Madrid, and Andalusia.

*FPs inclusion criteria:*

- Stable work situation, without intention to leave the position during the study.
- Agree to participate and sign the informed consent form

*Patient selection criteria:*

Inclusion criteria:

- Age: 65–74 years
- Multimorbidity: ≥ 3 chronic diseases
- Polypharmacy: ≥ 5 drugs prescribed for at least three months prior to inclusion in the study
- Having visited/contacted their family physician at least once in the last year
- Able to follow the requirements of the study
- Agree to participate and sign the informed consent form

Exclusion criteria:

- Institutionalized patients
- Life expectancy of less than 12 months based on their doctor
- Physical or mental illness that in the opinion of their doctor does not allow them to follow the study requirements.

### Sample size

It was calculated under the hypothesis that the intervention would result in a difference of at least six percentage points in the combined variable (hospitalizations and/or all-cause mortality) between the study groups. According to previous studies, in the age range of 65 to 75 years, the criterion incidence of the estimated primary outcome variable was 16% [23, 34]. Considering a power of 80%, an alpha error of 5% and assuming simple random sampling, the required sample size would be 984 patients (492 patients per group).

The appropriate sample size for this type of design depends on the average size of the cluster and the degree of correlation between the individuals in the cluster. Consequently, it is necessary to adjust the sample size calculated according to the design effect (DE). An average group size of eight patients per FP and an intraclass correlation coefficient of 0.02 [35] produces the following (DE = 1 + (5–1) × 0.02 = 1.08), giving a sample size, corrected for design effect, of 1063 patients. Assuming a loss rate of 8%, the final sample size required is 1148 patients (574 per group).

### Recruitment

MULTIPAP Plus has a group of 120 FPs who previously participated in the MULTIPAP project; voluntary participation has been proposed to this group and another FPs working in PC health centres in Aragón, Madrid, and Andalusia. Strategies to improve protocol adherence of FP will be considered (e.g. individual follow-up of protocol’s achievements and recognition via e-mail, offer to participate as co-authors in scientific papers, certified training sessions).

**Fig. 1:**
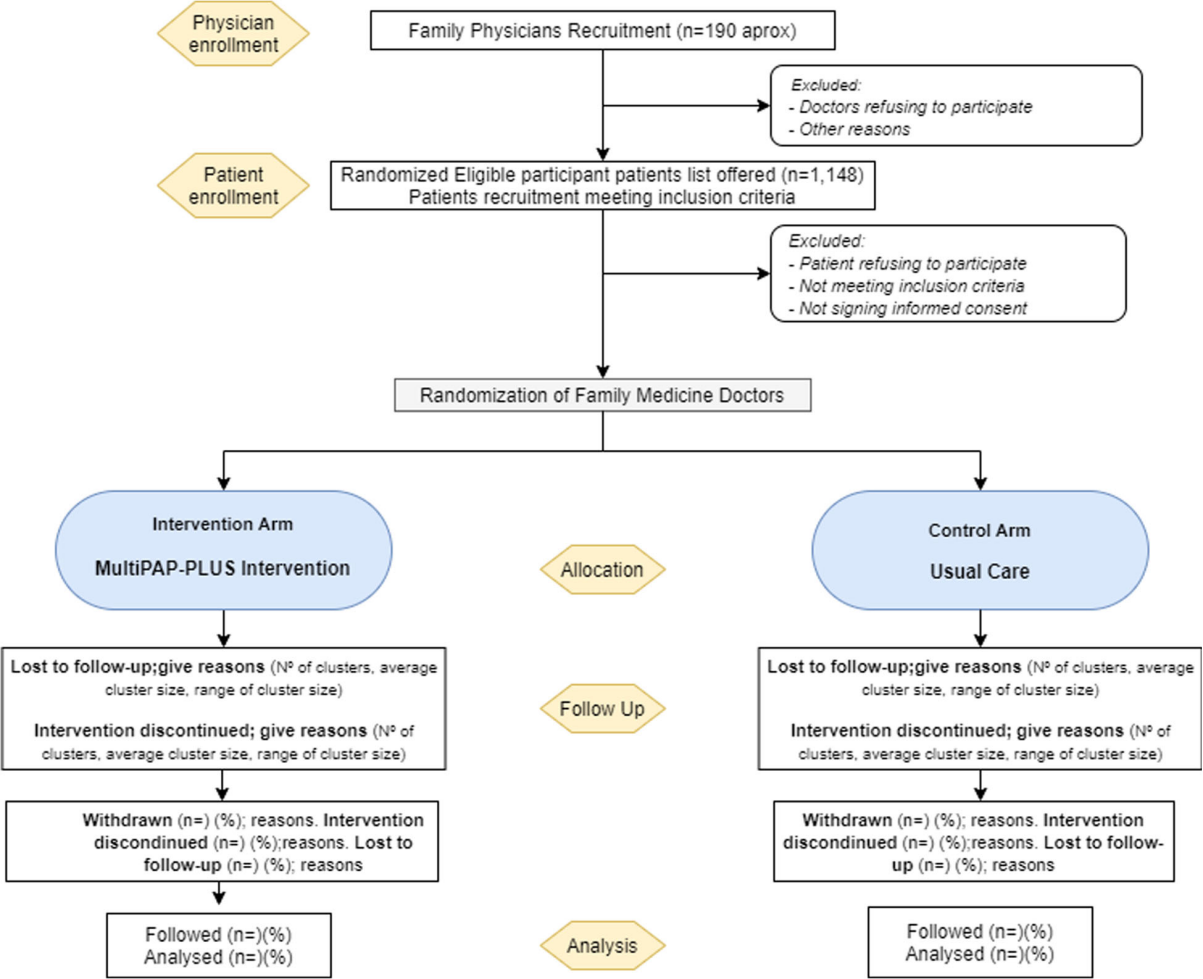
Flow diagram of patients

Patients will be selected by random sampling from the list of patients who meet the inclusion criteria. Subsequently, each FP will invite the listed patients, when the patient agrees to participate, the FP will inform them in detail about the study and confirm the inclusion/exclusion criteria and obtain the patient’s written informed consent. If they do not agree to participate, data on the patient’s age, sex, and reason for nonparticipation will be collected (see Fig. 1, flowchart).

The selection of professionals was carried out in the third quarter of 2019. The recruitment of patients began in November 2019 and was suspended in March 2020. Considering the epidemiological situation in Spain, and the reorganization of health services including primary care, it was not possible to restart the study until May 2021. In that month, the situation of the family physicians was reviewed, and the commitment was updated, as well as the situation of the patients recruited between November 2019 and February 2020.

### Randomization

The unit of randomization is the FP, and the unit of analysis is the patient. The randomization of FPs will be achieved using the treatment assignment module of Epidat® 4.1; the proposed intervention will be considered the treatment, and usual care will be considered the control. To guarantee an equal number of FPs in each group (intervention and control), the option “balanced groups” will be selected. Once all participating FPs have selected their patients and have collected the corresponding initial data, the Research Unit, Primary Care Management of Madrid, will randomize them centrally. Subsequently, each FP will receive the information of the study group to which they have been assigned, at which time all the patients they have recruited will be included in that group.

### Intervention

This is a complex intervention that includes the training of FPs and physician-patient interview based on the Ariadne principles; the effectiveness of the intervention has been studied in the MULTIPAP RCT [23, 36, 37]. The training includes activities related to basic concepts of multimorbidity, the appropriateness of prescriptions, adherence to treatment, the ARIADNE principles, and shared doctor-patient decision-making. All participating physicians will receive this training with the objective of incorporating patient-centred interviews into their routine clinical practice. This would allow the effect of the training to be isolated from the use of a DSS.

#### Intervention group

Several key elements of the intervention must be highlighted:

1. Clinical data will be reviewed and registered by FPs in the electronic case report forms (eCRF) after recruitment in baseline visit.
2. Because of the natural separation between actual electronic health records and electronic case report forms developed for the research project, final tool has been integrated into a dedicated eCRF system using a webservice WS4 version 1.2 of the CheckTheMeds® tool with the specific inputs reviewed by the FPs. This avoids barriers for FPs and the need for reintroducing double information to a separate system. Simple and detailed review of treatment plans has been incorporated. Simple review webservice outputs have been agreed after a Delphi technique with relevant stakeholders (primary care pharmacist, hospital pharmacist, family physicians, and researchers).
3. The web-based, user-initiated DSS provide FPs with drug-therapy information that is relevant to participating patients with polypharmacy on demand. After verifying the clinical data included in the eCRF (multimorbidity and polypharmacy corrected for patient-specific factors such as sex, renal function, age and frailty), this DSS provides health professionals with clinical scenarios to optimize treatment plans for patients, the number of STOPP/START and Beers criteria, drug interactions classified based on their potential severity (traffic light system), and adjustment of the medications to other relevant clinical variables.
4. FPs will be able to add and modify patient data in eCRF and able to review again treatment plans as many times as needed with up-to-date relevant information (new drugs, new diagnoses, change if laboratory findings about kidney function, etc.).
5. A specific training tool with video-recorded examples for clinical scenarios with treatment plans reviews appearing on first access to the tool and available for revising when needed as part of the formative intervention.

**Fig. 2:**
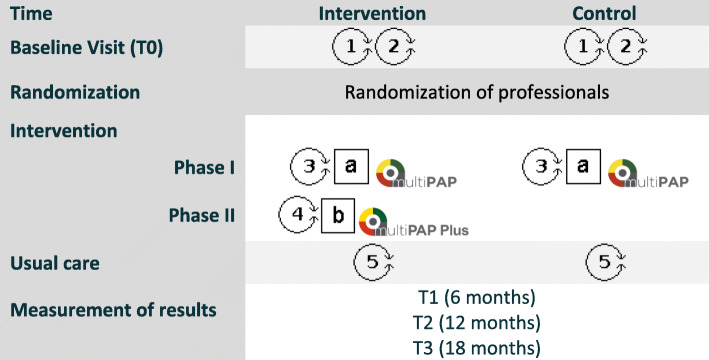
Intervention

There are no restrictions regarding concomitant care during the trial. This intervention was developed in accordance with the recommendations and the taxonomy proposed by the Cochrane Effective Practice and Organization of Care Group (EPOC). The intervention is described in detail in Fig. 2, following the approach proposed by Perera et al. and the Template for Intervention Description and Replication (TIDieR) (Additional file 3) [38]..

### Control group

The patients in the control group will receive the usual clinical care based on the provision of advice and information and will be subjected to the examinations recommended in the guideline corresponding to each of the chronic diseases presented by the patient. Physicians will receive the same initial training programme as professionals in the intervention group.

### Blinding

Due to the type of intervention, neither FPs and their patients nor the MULTIPAP Plus study team was blinded to the treatment allocation.

### Variables

FPs will provide their data before the start of the study. Patient data will be collected by the recruiting FP, who will also be responsible for patient follow-up. All information will be recorded in a case report form designed for the study. Each FP will access the form from their personal computer through the project website using a personal identification code. Four visits were defined for patient data collection: baseline (T0), 6 months (T1), 12 months (T2), and 18 months (T3) (see Table 1).

**Table 1:** Visit plan

	T0 (baseline)		T1 (6 m*)	T2 (12 m*)	T3 (18 m*)	Responsible entity
Confirm inclusion/exclusion criteria	X					FP
Written informed consent	X					FP
Sociodemographic variables	X					FP
Morbidity variables and drug treatment plan	X		X	X	X	FP
Randomization of FP´s		X				RU
FP intervention (intervention group)		X				RT
Patient intervention (intervention group)			X	X	X	FP
Mortality				X	X	RT/FP
Hospitalizations				X	X	RT/FP
Functionality (WHODAS), falls, hip fractures			X	X	X	FP
Use of health services			X	X	X	RT
Quality of life	X			X	X	FP
Usability, adhesion, satisfaction			X		X	TG
Costs					X	RT

*T* time, *FP* family physician, *RU* research unit, *IG* intervention group, *RT* research team, *TG* technical group, m* months from randomization

#### Primary outcome variable

The primary outcome is a composite endpoint of hospital admission or death during the observation period measured as a binary outcome. Primary outcome measures are recorded by the FPs when they occur or at follow-up visits.

#### Secondary outcome variables

- All cause mortality, non-elective hospital admission (number of episodes and duration)
- Related to the use of medication: Potentially inappropriate prescription will be evaluated in accordance with the BEER criteria [39] and the STOPP-START criteria [40]; medication safety will be measured as the incidence of adverse reactions and potentially dangerous interactions, classified based on the taxonomy proposed by Otero-López [41] as well as the incidence of adverse reactions; and adherence to treatment will be measured. Medication adherence was measured with the Morisky Medication Adherence score [42].
- Use of health services will be measured using records of unplanned and/or preventable hospitalizations as well as the use of emergency services and PC (FP and nurse).
- Quality of life will be measured using the EQ-5D-5L questionnaire [43, 44]. The differences in utility index between the intervention group and the control group at 18 months of follow-up will be used to determine the QALYs gained due to the intervention. The scores or utilities derived from the latest version of this tool, the EQ-5D-5L, have been proposed to provide information on economic evaluations of technologies.
- Disability will be measured using the WHODAS questionnaire [45] and number of fractures.
- For cost-utility, the National Health Service perspective will be adopted, with a time horizon of 2 years and a discount rate of 3%. The costs incurred will be the time dedicated to training required by the training programmes, the cost of the teaching staff, the time dedicated to doctor-patient interviews, and the rights to use of the tool (DSS). All costs derived from the intervention will be charged through an opportunity cost proxy: average salary by professional category. As “avoided costs” of the intervention, we will consider the price of the drugs discontinued (measured using the retail price) and the cost of the adverse reactions avoided. The EQ-5D-5L questionnaire will be used to estimate the utilities in both groups (intervention and control). The QALYs obtained with the intervention will be calculated and compared with the difference between the costs incurred and avoided.
- DSS usability will be measured from user analysis perspective and usability testing [46] to the direct users (FPs): (a) User analysis: this analyses user behaviour while interacting with a website. It is going to be realized non-obtrusively in the background by recording mouse movements and click behaviour to identify to what extent functions and website areas are accessed when reviewing treatments (Hotjar Tool); (b) usability using an ad hoc design with the Spanish-validated System Usability Scale (SUS) [47] and Computer Software Usability Questionnaire [48], DSS acceptability will be measured using records of actions performed with the CheckTheMeds® algorithm (number of changes) and a review of time invested per professional, and DSS satisfaction will be measured using an ad hoc questionnaire.

#### Explanatory and adjustment variables

a) Patient variables (first level)

- Sociodemographic: age, sex, nationality, region of residence, marital status, socioeconomic status (monthly salary expressed as multiples of minimum wage), family composition (number of people living in the household), indicators of subjective urban vulnerability, based on those collected by the National Health Survey to explore participants’ neighbourhood (noise level, odours, poor-quality drinking water, unclean streets, air pollution, lack of green areas, feral animals and crime), social support (Duke-UNC-11 Questionnaire adapted to Spanish [49]), profession, and social class [50].
- Morbidity: number and description of chronic diseases based on the International Classification of Diseases in Primary Care (ICPC) as per O’Halloran list [51].
- Pharmacotherapeutic treatment plan: number and type of drugs prescribed and active ingredient and dose of each drug.

b) FP variables (second level)

- Sociodemographic: age and sex.
- Professional performance: years of professional experience, resident mentor (yes/no), and average workload measured as average daily consultations per FP during the year prior to the start of the study.

### Oversight and monitoring

#### Roles and composition of the trial management committee and trial steering committee

##### Principal investigators

Each participating region will have a lead investigator who will be the senior researcher receiving the public funding and will be responsible for identification and physician recruitment. Principal investigators will be steering committee members.

##### Research physicians

Any of the physician recruiting patients involved in the data collection and completion of CRFs, along with the follow-up of study patients and adherence to study protocol and investigators brochure.

##### Trial steering committee (TSC) (see title page for members)

All principal investigators will be steering committee members. Aragon region principal investigator will be national coordinator. Tasks: Agreement of final protocol. Recruitment of patients and liaising with principle investigator (PI). Reviewing progress of study and if necessary agreeing changes to the protocol and/or investigators brochure to facilitate the smooth running of the study.

##### Trial management committee (TMC)

There will be a principal investigator, a research physician, a statistician, and a administrator at each region. Tasks are as follows: study planning, organization of steering committee meetings, provide annual risk report and ethics committee serious unexpected suspected adverse events, responsible for trial master file, budget administration and contractual issues with individual centres, advice for lead investigators, audit of 6 monthly feedback forms and decide when site visit to occur, assistance with international review and board/independent ethics committee applications, data verification, and randomization (this in Madrid Region).

##### Data manager

There will be one per each region. Tasks are as follows: maintenance of trial IT system and data entry and data verification.

#### Composition of the data monitoring committee, its role, and reporting structure

A data monitoring committee (DMC) has been established based on the vital-status outcome measurement chosen and the long trial duration (18 months). The DMC is independent of the study organizers. For the data monitoring committee proposal, we have taken into account the distinguishing characteristics of pragmatic clinical trials [52–54]. This committee includes clinicians, biostatisticians, and ethical experts, and given the patient-centred outcomes focus of our trials [55, 56], a patient representative is incorporated to provide patient’s perspective. This will allow to review relative benefits, burdens, and potential harms of the interventions and will provide insight into the optimal ways for results sharing [57, 58] to participants and relevant patient group.

During the period of recruitment to the study, interim analyses will be supplied, in strict confidence, to the DMC, together with any other analyses that the committee may request. This may include analyses of data from other comparable trials. In the light of these interim analyses, the DMC will advise the TSC. The frequency of interim analyses will depend on the judgement of the Chair of the DMC, in consultation with the TSC. However, we anticipate that there might be one interim analysis at 12 months after randomization and one final analysis at 18 months. The interim analysis will be performed by an independent statistician, blinded for the treatment allocation. The statistician will report to the independent DMC.

### Statistical analysis

All analyses will be carried out according to the intention-to-treat principle, with a statistical significance at *p* < 0.05.

Description of the baseline characteristics means and standard deviations for the quantitative variables and absolute and relative frequencies for the qualitative variables, with their corresponding 95% confidence intervals (95% CIs). Likewise, the characteristics of those patients who leave the study will be described, including the reason for loss during follow-up.

Basal comparisons between groups will be performed using statistical tests for independent samples (Student’s *t*-test or chi-squared test). Tests for related samples (ANOVA for repeated measures) will be used to analyse changes within groups and between visits.

#### Analysis of main effectiveness

The difference in percentages in the composite endpoint of hospital admission or death to 18 months with its corresponding 95% CI. Multilevel analysis will be adjusted considering the combined final variable as the dependent variable; the baseline variables of the patient (first level), the professional (second level), and the intervention group as independent fixed effect variables; and the clustering by physician as a random factor. We will use multiple imputation by chain equations including baseline, 6-month, and 12-month data as available, intervention group, stratification and minimization variables, and other covariates that were informative of missingness. A sensitivity analysis will be conducted to assess whether conclusions may change if assumptions about missing data change.

#### Analysis of secondary effectiveness

The same analysis will be performed for 12 months. Missing data for professionals and/or patients will be replaced with the most recent reference or available data. For the remaining secondary effectiveness analyses, the difference in means or proportions will be calculated based on the characteristics of the variables (T3−T0) and (T2−T0) between groups using the appropriate statistical tests, and an explanatory model will be adjusted using the same methodology applied to the main outcome variable.

*Estimated quality-adjusted life years (QALYs)* gained at the population level, with corresponding 95% CI, as determined using parametric methods and bootstrap techniques. Given the 2-year time horizon, a discount rate of 3% will be applied.

#### Calculation of the cost-utility ratio

A cost-utility ratio will be estimated by dividing the total costs by the sum of the potential gains expressed in QALYs. A multivariate sensitivity analysis will be performed by varying the value of the costs with the appropriate distributions within the range of uncertainty. The benefits (QALYs) will also vary with the most suitable distribution in the 95% CIs of the estimates. It will also be included in the sensitivity analysis.

#### User analysis and usability testing analysis

Descriptive statistics will be used to analyse CSUQ an SUS scores according to the formal way of analysis to compare between participant groups (FPs) to take into account gender and age differences [59] in usability. No repeated-measurements neither pre-post test will be analysed.

## Discussion

This pragmatic clinical trial will involve the participation of FPs from over 130 PC health centres in different geographic areas of Spain, thus ensuring a high level of external validity, given that the PC model implemented throughout the country is relatively homogeneous.

Among the possible limitations or biases of our study, we take into consideration that professionals who agree to participate may be more motivated, leading to observer bias (Hawthorne effect). Those professionals more involved in the work will probably make greater use of the DSS than if it were established under normal conditions. If the tool is effective, we might observe an overestimation of the effect that if applied to routine clinical practice would be more limited. This could lead to a greater effectiveness than would be obtained under normal conditions, but it is expected that when randomization is performed after the inclusion of patients in the study, the magnitude of the difference between the two groups will be minimized. Additionally, all physicians will have previously received the training intervention. In this way, it will be ensured that all physicians receive the same training and share the same knowledge of the study, thus reducing performance bias.

The use of a composite endpoint as hospital admission or death is proposed as recommended by some European studies, in order to compare the results with these studies [28, 60, 61].

The intervention cannot be blinded, which could influence the results. However, because the variables can be obtained from records, those who carry out the analysis and their interpretation will not know to which group the patient belongs. In turn, to avoid possible contamination of the professionals in the control group, the intervention group will be advised of their commitment to confidentiality. The control group may use the DSS to review the treatments of the patients included in the study at the end of follow-up for a similar period of time. The expected changes in quality of life are small, but their measurement allows incorporating outcome variables reported by the patient and calculating utilities.

## Trial status

Protocol Version Rev.0 (19.03.2019) + Anexo 2 Rev.0 (05.04.2019). Recruitment started on February 27, 2020. Approximate date for end of recruitment February 28, 2022.

## Supplementary Information

**Additional file 1:** CONSERVE-SPIRIT checklist.

**Additional file 2:** SPIRIT Trial Modifications in Extenuating Circumstance.

**Additional file 3:** TIDieR.

## Data Availability

The data that support the findings of this study will be available from research unit uinvestigación.ap@salud.madrid.org, but restrictions apply to the availability of these data, which were used under licence for the current study, and so are not publicly available. However, each new project based on these data must be previously submitted to CEICA for approval. Data will be however available from the authors upon reasonable request and with permission of the project’s principal investigators (Alexandra Prados-Torres at sprados.iacs@aragon.es; Daniel Prados-Torres at uand.prados.sspa@juntadeandalucia.es; and Isabel del Cura at isabel.cura@salud.madrid.org). The datasets analysed during the current study and statistical code are available from the corresponding author on reasonable request, as is the full protocol.

## Abbreviations

FP: Family physician
PC: Primary care
SR: Systematic review
ICT: Information and Communication Technologies
FPs: Family physicians
DSS: Decision support system
eCRF: Electronic case report form
QALY: Quality-adjusted life year
PIP: Potentially inappropriate prescription
