# Supplementary material for: Effectiveness of the MULTIPAP Plus intervention in youngest-old patients with multimorbidity and polypharmacy aimed at improving prescribing practices in primary care: study protocol of a cluster randomized trial
Source: Trials. 2022 Jun 9;23:479. doi: 10.1186/s13063-022-06293-x (PMC9178530; doi:10.1186/s13063-022-06293-x)
Supplement: Supplementary file 1 — Additional file 1: CONSERVE-SPIRIT checklist. [file 13063_2022_6293_MOESM1_ESM.pdf]

CONSERVE-SPIRIT Extension: [DATE]					
**Item**	**Item Title**	**Description**			**Page No.**
I.	Extenuating Circumstances	Describe the circumstances and how they constitute extenuating circumstances.			6
II.	Important Modifications	a. Describe how the modifications are important modifications.			sup 2, 3
		b. Describe the impacts and mitigating strategies, including their rationale and implications for the trial.			(see below)
		c. Provide a modification timeline.			sup 2,3
III.	Responsible Parties	State who planned, reviewed and approved the modifications.			PIs; Sponsor, Eihc Comitte
IV.	Interim data	If modifications were informed by trial data, describe how the interim data were used, including whether they were examined by study group, and whether the individuals reviewing the data were blinded to the treatment allocation.			
**SPIRIT Item and Number**		For each row, if important modifications occurred, check one or both of "impact" and/or "mitigating strategy" and describe the changes in the protocol. Check "no change" for items that are unaffected in the extenuating circumstance.			**Page No.**
		**No Change**	**Impact***	**Mitigating Strategy****	
1	Title	No Change			
2	Trial registration	No Change			
3	Protocol version	Change	version with changes	Publish study protocol with modifications	1-15
4	Funding	No Change			
5	Roles and responsibilities	No Change			
6	Background and rationale	No Change			
7	Objectives	No Change			
8	Trial design	No Change			
9	Study setting	No Change			
10	Eligibility criteria				
11	Interventions	No Change			
12	Outcomes	No Change			

13	Participant timeline	change			
14	Sample size	change	Each physician will be able to recruit fewer patients, from 8 to 5.	Decreases cluster size, design effect, sample size	7
15	Recruitment	change	New recruitment period, with longer duration.	Until December 2021	8
16	Allocation	No Change			
17	Blinding (masking)	No Change			
18	Data collection methods	No Change			
19	Data management				
20	Statistical methods	No Change			
21	Data monitoring	No Change			
22	Harms	No Change			
23	Auditing	No Change			
24	Research ethics approval	No Change			
25	Protocol amendments				
26	Consent or assent	No Change			
27	Confidentiality	No Change			
28	Declaration of interests	No Change			
29	Access to data				
30	Ancillary and post-trial care	No Change			
31	Dissemination policy	No Change			
32	Informed consent materials	No Change			
33	Biological specimens	not applicable			

*Aspects of the trial that are directly affected or changed by the extenuating circumstance and are not under the control of investigators, sponsor or funder.
**Aspects of the trial that are modified by the study investigators, sponsor or funder to respond to the extenuating circumstance or manage the direct impacts on the trial.

The CONSERVE-SPIRIT Checklist is licensed by the CONSERVE Group under the Creative Commons Attribution-NonCommercial-NoDerivs 4.0 International license.
